# Long term trends in behaviour to protect against adverse reproductive and sexual health outcomes among young single African women

**DOI:** 10.1186/s12978-018-0576-6

**Published:** 2018-08-14

**Authors:** Mohamed M. Ali, John Cleland

**Affiliations:** 1 UNDP-UNFPA-UNICEF-WHO-World Bank Special Programme of Research, Development and Research Training in Human Reproduction (HRP), World Health Organization, 20 Avenue Appia, 1211 Geneva, Switzerland; 2 Department of Population Health, Faculty of Epidemiology and Population Health, London School of Hygiene and Tropical Medicine, Keppel Street, London, WC1E 7HT UK

## Abstract

**Background:**

HIV and unintended pregnancy are major interrelated concerns in sub-Saharan Africa. Focussing on single women aged 15-24 years we assess trends in key behaviours that affect both outcomes.

**Methods:**

We performed a secondary analysis of public-access data sets from 112 surveys from 36 countries in the region, conducted between 1991 and 2015. We examined trends over 20 years in primary abstinence (virginity), secondary abstinence (no sex in past 3 months) among sexually experienced women, current use of modern contraception and condom use at most recent coitus among sexually active women.

**Results:**

Little change occurred in primary or secondary abstinence. Over the 20 year period, contraceptive use in the region rose from 14.7 to 33.4%, with significant increases observed in 18 of 30 countries with multiple surveys. Since 2001–2005, the proportion of contraceptive users reporting condoms as their method fell from 61.1 to 51.3%, while use of oral contraceptives or injectables rose from 19.9 to 24.0%. Between 1996 and 2000 and 2006–2010, condom use at last coitus rose from 21.3 to 40.5% but then plateaued. A strong correlation between condom use and national HIV prevalence was found. About half of condom users at last sex had earlier in interviews reported this method for pregnancy-prevention.

**Conclusions:**

Though condoms tend to be overlooked by both HIV and family planning agencies, their contribution to the health of single women remains central. Current efforts to promote non-barrier contraceptive methods may inadvertently increase HIV risk. Condom promotion for pregnancy-prevention should be re-invigorated by social marketing campaigns and other means.

**Electronic supplementary material:**

The online version of this article (10.1186/s12978-018-0576-6) contains supplementary material, which is available to authorized users.

## Plain English summary

Using data from 112 surveys conducted in 36 countries, we examine trends over the past 20 years in behaviours that protect against pregnancy and sexually transmitted infections. We focus on single women aged 15 to 24 years living in sub-Saharan Africa. About half of these young women reported no experience of sexual intercourse (ie virginity). Sexual encounters are infrequent for many: among non-virgins, only about half had sex in the 3 months before they were interviewed. Abstinence is clearly a major form of protection but has changed little over the 20 year period, suggesting that campaigns to promote it as a primary defence against HIV were largely ineffective. Trends in current use of contraception and condom use at most recent sex are encouraging. In 2011–5, one-third of women with sexual activity in the past 3 months said that they were using a contraceptive method and 42% reported using a condom at last sex. The proportion of contraceptive users relying on condoms has recently dipped from 61 to 51% while those relying on pills or injectables grew to 24%. We conclude that condoms remain the optimal method of contraception for young women for whom sex is infrequent and loving in localities the risk of infection is high. We call for a re-invigoration of condom social marketing. While non-barrier contraceptive methods are appropriate for single women in more committed and intimate relationships, promotion should be cautious because their high contraceptive effectiveness has to be balanced against their lack of protection against infection.

## Background

The health of young people, aged 15-24 years, has become a global priority, as demonstrated by the UN’s Global Strategy for Women’s, Children’s and Adolescent’s Health [1], reports from the UN Population Fund [2], the creation of a Lancet Commission in 2012, and innumerable other international declarations. Prominent among concerns are reproductive and sexual health, particularly in sub-Saharan Africa (henceforth Africa). Because of the combination of high birth rates and declining mortality, the youth population in this region is particularly large, accounting for 38% of the total. Rates of teenage childbearing and HIV/STI infections are much higher than in all other regions. Maternal mortality ratios in young women and abortion rates in unmarried women are estimated to be higher in Africa than elsewhere and, because of restrictive laws, many abortions are unsafe [3, 4].

A very large literature has accumulated on the reproductive and sexual health, and associated behaviour, of young women in Africa. Age at first marriage, or cohabitation, has increased [5, 6]. Age at first birth has also risen, due in large measure to improvements in girls’ schooling [7]. The level of premarital sex has risen despite a trend towards delayed sexual debut [7, 8]. In some countries premarital childbearing has increased while in others it has declined or remained unchanged [5]. Contraceptive use among sexually active unmarried teenagers has increased though contraceptive failure and discontinuation are more common in this group than among older women [9]. Unmet need for contraception--non-use in those wanting to avoid pregnancy-- in African adolescents remains high [10].

Trends in HIV incidence are encouraging with a 42% decline of new infections among youth in Africa between 2001 and 2013 [11]. However, young women remain at much higher risk than young men and account for 25% of all new infections in adults. Several favourable trends have contributed to this decline and relevant information is available from the electronic data base of the Demographic and Health Survey (DHS) program [12]. Reported condom use at most recent coitus among those with a recent non-cohabiting partner, or with multiple partners, has increased in many countries though the level remains unsatisfactory. The percentage of sexually active young women who received an HIV test in the past 12 months and were told the results has increased greatly in East and Southern Africa. In 14 priority countries, 11.6 million men have been circumcised [11]. DHS data confirm the upward trend in circumcision [12].

In this paper we assess behavioural change by never married African women aged 15-24 years. Specifically, we analyse trends in abstinence, current contraceptive use and condom use at most recent sex drawing on all DHSs conducted since 1991. The inclusion of information on multiple partners would have provided a more comprehensive assessment of behavioural change but we decided against it because of convincing evidence that this information is particularly unreliable [13]. Our paper complements the extensive existing literature in several ways. First, we present a clear chronological record of change, starting with data from 1991 to 1995 and ending with the most recent quinquennium, 2011–2015. Second, we pay equal attention to pregnancy- and HIV-prevention in contrast to most publications that focus on only one of these intertwined behaviours. Third we examine changes in type of contraceptive method used and in double method protection. Fourth, we estimate the effect of structural shifts in the study population over the 20 year period, namely age, education and rural-urban residence, on reported behavioural change.

## Methods

The main methods used in this paper were described in an earlier publication [14]. In brief, we downloaded the individual data files from all DHSs conducted in Africa from 1991 to 2015 that are publically available on 31 December 2016. A total of 112 surveys from 36 countries were used. The analysis population comprised single (i.e. never married) women aged 15-24 years. We focussed on 4 self-reported behavioural outcomes: primary abstinence (or virginity); secondary or temporary abstinence in sexually experienced women (defined as no intercourse in the 3 months preceding the survey); current use of modern contraceptive methods among women who had been sexually active in the 3 months preceding the survey; and condom use at most recent coitus among the sexually active. We chose a 3 month boundary for secondary abstinence rather than the more commonly used 12 month definition because of doubts about the meaning of reported “current” condom use by women who have been sexually inactive for several months. Estimates of these four outcomes were calculated for denominators with 100 or more women. Sample sizes and denominators for all 112 surveys are shown in Additional file 1.

To assess trends we grouped all surveys by date of fieldwork as follows: 199 1-95, 1996–2000, 2001–05, 2006–10, and 2011–2015. The number of surveys in each 5 year period ranged from 15 in the first period to 28 in the last. Estimates from individual surveys in each period are summarised by box plots, showing medians and inter-quartile ranges (IQRs). Exact 95% CIs for the median values were calculated from the binomial distribution. We were unable to estimate condom use last coitus for the period 1991–1995, owing to insufficient number of surveys with the relevant information.

We also present country-specific results in Additional files 1, 2, 3, 4, 5, 6, 7, 8 and 9. For the 30 countries with multiple surveys, we calculated the median date of fieldwork for first and most recent survey and estimated the annual rate of change in the prevalence of each of the 4 outcomes. We assessed the significance of within-country changes using logistic regression by including a binary indicator for survey round with robust standard errors. In addition to the crude percentages, standardized percentages were also computed, using the composition of the earliest survey for each country with respect to age (in 3 year bands), place of residence, education (primary school or less versus secondary or higher) as the standard population.

To assess whether trends had been distorted by variations in the countries represented in each time period, we re-did the analyses for the 16 countries with 4 or more DHSs. The comparison, shown in Additional file 2, showed a close similarity between all-survey and multiple surveys results. The survey-normalized weights were applied to take account of variations in probabilities of selection corrected for non-response. All analyses were carried out in Stata 14.2.

## Results

### Abstinence

**Fig. 1:**
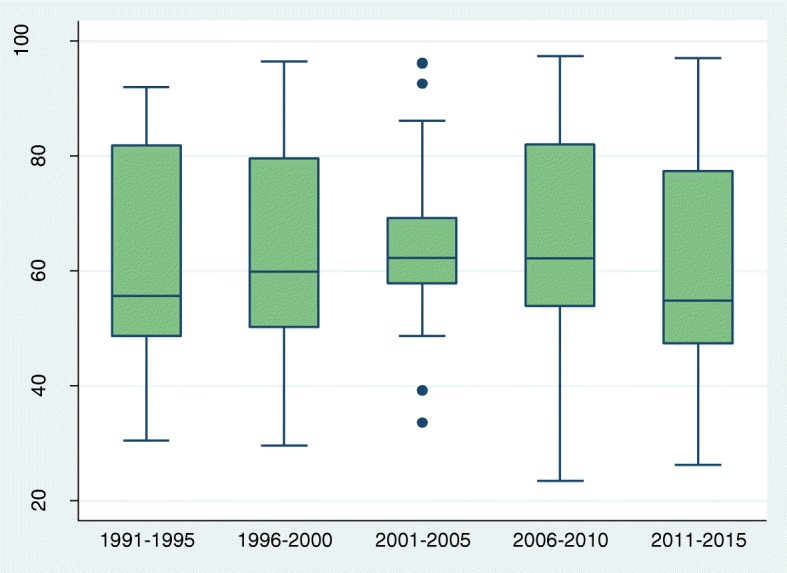
Box and whisker plot showing trend (1991–2015) in the percentage of single women aged 15–24 years who reported no sexual experience

Trends in primary abstinence (virginity) were modest and irregular, ranging from 62.3% (95%CI: 57. 9-68.7) in 200 1-5 to 54.8% (95%CI: 49. 1-67.9) in 201 1-5 with no statistically significant changes (Fig. 1 and Table 1). The inter-quartile ranges (IQRs) were wide in all periods except 200 1-2005, indicating large and persistent variability between countries. Of the 30 countries with two or more surveys, significant (*p* < 0.01) increases are apparent in six, notably Madagascar and Malawi, and significant decreases in four, notably Guinea, Lesotho and Rwanda (Additional file 3).

**Table 1:** Medians and 95%CIs for primary abstinence (virginity), secondary abstinence (no sex in past 3 months), current use of modern contraception and condom use at last sex: crude and adjusted percentages, by period

Period	No of surveys	Crude		Adjusted	
		Median	(95%, CI)	Median	(95%, CI)
Primary abstinence					
1991–1995	15	55.6	(48.8, 81.2)	55.6	(48.8, 81.2)
1996–2000	25	59.8	(52.8, 73.0)	59.8	(52.7, 72.9)
2001–2005	21	62.3	(57.9, 68.7)	62.6	(57.7, 71.4)
2006–2010	23	62.1	(54.3, 73.2)	61.0	(56.9, 77.2)
2011–2015	28	54.8	(49.1, 67.9)	58.4	(51.7, 68.6)
Secondary abstinence					
1991–1995	15	51.1	(35.8, 58.9)	51.1	(35.8, 58.9)
1996–2000	23	38.3	(35.1, 48.7)	38.3	(35.1, 50.4)
2001–2005	21	48.4	(42.1, 62.8)	47.2	(41.8, 62.8)
2006–2010	22	44.6	(41.7, 60.3)	45.4	(41.5, 60.5)
2011–2015	26	44.0	(38.7, 56.7)	44.9	(39.9, 57.1)
Current use of modern contraception					
1991–1995	11	14.7	(5.1, 26.5)	14.7	(5.1, 26.5)
1996–2000	22	23.2	(19.1, 29.9)	22.5	(16.0, 28.4)
2001–2005	17	30.2	(23.0, 42.1)	27.9	(19.7, 37.4)
2006–2010	20	31.6	(27.3, 39.6)	29.8	(21.5, 36.7)
2011–2015	24	33.4	(28.5, 46.6)	31.2	(27.0, 41.5)
Condom use last sex					
1991–1995		NE		NE	
1996–2000	21	21.3	(15.3, 26.5)	21.9	(13.2, 25.1)
2001–2005	17	33.6	(25.4, 42.5)	29.2	(26.2, 37.1)
2006–2010	20	40.5	(29.5, 50.6)	37.4	(23.9, 44.9)
2011–2015	24	41.9	(34.8, 49.7)	39.6	(29.7, 48.4)

*NE* Not Estimated due to few number of surveys

**Fig. 2:**
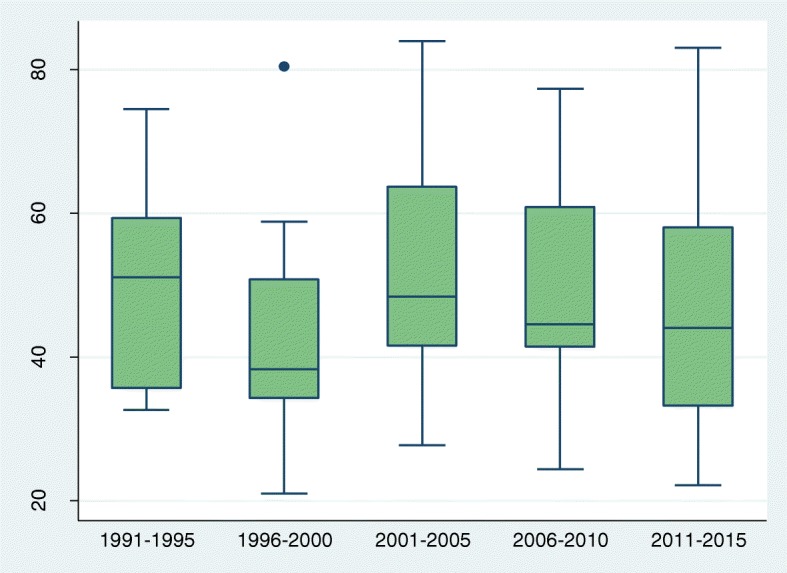
Box and whisker plot showing trend (1991–2015) in the percentage of sexually experienced single women aged 15–24 years who reported no sex in the 3 months before the survey

Trends in secondary abstinence were also irregular and non-significant, ranging from 48.4% (95%CI: 42.1–62.8) to 48.4% (95%CI: 42.1–62.8) in different periods (Fig. 2 and Table 1). Statistically significant changes were apparent in only 6 countries (Additional file 3). Levels of secondary abstinence are high and, in conjunction with with primary abstinence suggest that, on average, only about 25% of young women were exposed to potential risk in the past 3 months.

### Contraception

**Fig. 3:**
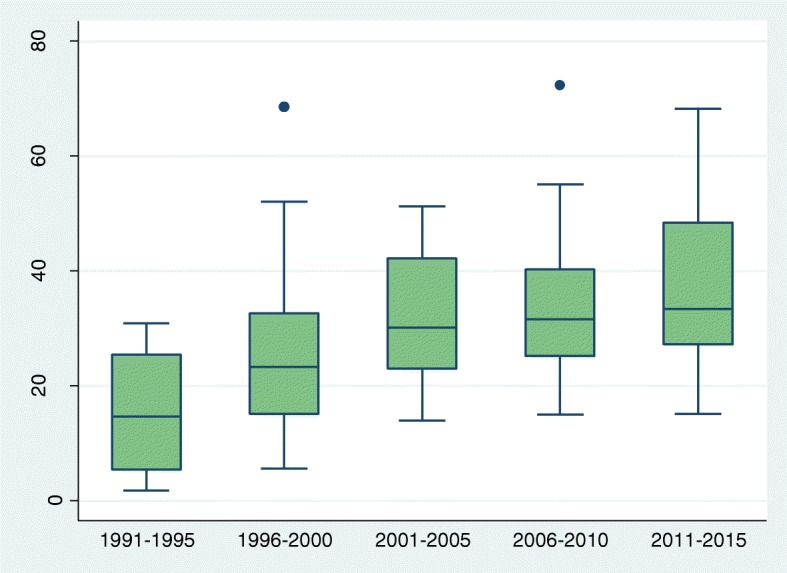
Box and whisker plot showing trend (1991–2015) in percentage who reported current use of a modern contraceptive method, among single women aged 15–24 who had sex in the last 3 months

The median level of current use of a modern method of contraception among women who had sex in the past 3 months rose sharply and significantly from 14.7% (95%CI: 5.1–26.5) in the early 1990s to 33.4% (95%CI: 28. 5-46.6) in the most recent period. However, the big increase occurred between the early 1990s and the early 2000s and change since then have been modest have been modest (Fig. 3 and Table 1). A clear correlation with education only emerged in the past decade, rising to 0.64 (*p* < .0001) in the most recent quinquennium (Additional file 4). Inter-country variability changed little as indicated by the IQRs. Statistically significant increases are observed in 18 countries, and the mean annual increase exceeded 2% in Cameroon, Congo and Sierra Leone (Additional file 5). At the time of the most recent survey, current use of a modern method exceeded 50% in several West African countries (Cameroon, Nigeria, Sierra Leone) and several Southern African countries (Lesotho, Namibia, Republic of South Africa and Swaziland).

Examination of method-specific use, including traditional methods, revealed large temporal changes (Table 2). In 1991–1995, periodic abstinence was the most commonly reported method, accounting for 45.9% (95%CI: 20.0–69.7) of all reported use, but its contribution to protection declined sharply and monotonically over the 20 year period; in 2011–2015, the median percentage of users relying on this method was 6.4% (95%CI: 1. 4-15.0). The contribution of condoms to method-mix rose steeply from a median value of 25.9% (95%CI: 6. 2-34.0) in the early 1990s to 61.1% (95%CI: 47. 9-68.4) in 2001–2005 and 58.5% (95%CI: 45. 4-69.5) in the next quinquennium before falling to 51.3% (95%CI: 34. 8-65.9) in the most recent period. The role of oral contraceptives and injectables in contraceptive protection increased steadily, from a median value of 10.4% (95%CI: 5. 7-41.7) in the early 1990s to 24.0% (95%CI: 11.0–33.7) in the recent period. Use of long-acting methods, IUDs and implants, remained negligible.

**Table 2:** Median percentages using specific methods among current users of any method: single women aged 15-24 years, sexually active in the past 3 months

Period	Condoms	Oral contraceptives and injectables	Other modern methods	IUDs and implants	Periodic Abstinence	Other traditional methods
	Median (95% CI)	Median (95% CI)	Median (95% CI)	Median (95% CI)	Median (95% CI)	Median (95% CI)
1991–1995	25.9 (6.2, 34.0)	10.4 (5.7, 41.7)	0.3 (0.0, 1.1)	0.0 (0.0, 2.3)	45.9 (20.0, 69.7)	4.2 (1.2, 10.1)
1996–2000	35.2 (27.7, 47.7)	13.2 (10.9, 33.4)	0.0 (0.0, 0.8)	0.0 (0.0, 0.2)	25.2 (14.9, 48.3)	4.7 (2.5, 6.1)
2001–2005	61.1 (47.9, 68.4)	19.9 (12.8, 28.1)	0.4 (0.0, 0.7)	0.0 (0.0, 0.2)	14.0 (4.6, 29.0)	4.1 (1.8, 6.1)
2006–2010	58.5 (45.4, 69.5)	20.9 (15.5, 31.4)	0.4 (0.0, 1.5)	0.0 (0.0, 0.0)	4.9 (0.4, 13.2)	3.7 (1.7, 7.0)
2011–2015	51.3 (34.8, 65.9)	24.0 (11.0, 33.7)	2.4 (0.8, 9.5)	0.8 (0.0, 1.9)	6.4 (1.4, 15.0)	2.1 (1.4, 4.4)

### Condoms and dual protection

**Fig. 4:**
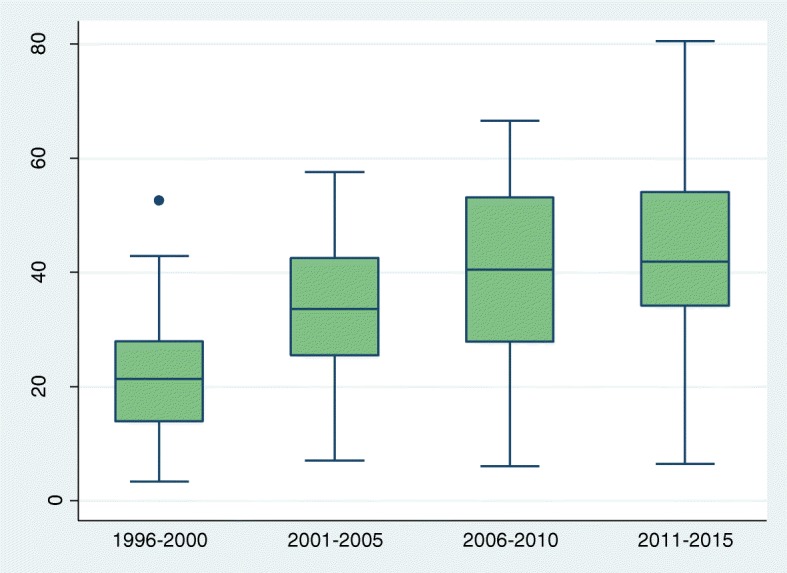
Box and whisker plot showing trend (1996–2015) in percentage who reported condom use at most recent coitus among single women aged 15–24 who had sex in the last 3 months

The median proportion of sexually active young women who reported condom use at most recent coitus rose significantly from 21.3% (95%CI: 15. 3-26.5) in 1996–2000 to 33.6% (95%CI: 25. 4-42.5) in 2001–5 and further to 40.5% (95%CI: 29. 5-50.6) in 2006–10, with an additional smaller increase to 41.9% (95%CI: 34. 8-49.7) in 2011–15. The IQRs are generally narrower than for other outcomes. The correlation between proportions using condoms and HIV prevalence among young people ages 15-24 years increased from 0.45 (*p* = 0.046) in 1996–2000 to 0.65 (*p* = 0.001) in the most recent period (Additional file 6). In more recent surveys, a question on consistent use was asked; on average, 74% of women who used a condom at last sex reported consistent use (Additional file 7). Reported use at most recent coitus was highest in Lesotho and Namibia, both countries with severe HIV epidemics, but was also over 60% in Burkina Faso, Cameroon and Gabon (Additional file 5). A total of 15 countries recorded significant increases in condom use and, in eight of them, the mean annual increase was 2% or more (Fig. 4).

Information about contraceptive use and condom use at most recent coitus are ascertained at different times in DHS interviews and the question on condom use at last coitus makes no mention of the motive for use. Cross tabulation can thus reveal useful insights into dual method protection and consistency of reported condom use. Table 3 shows the median percentages reporting condom use at most recent coitus by contraceptive use status (no method, condoms and non-barrier method). Among women who reported no contraceptive use, the proportion who used a condom at most recent coitus rose from about 7.9% (95%CI: 4. 8-12.4) in the 1996–2000 to about 30% in the past 10 years. Among users of modern or traditional non-barrier methods, reported use of condoms also rose from 15.3% (95%CI: 11. 3-23.9) in the 1996–2000 to 30.8% (95%CI: 27. 4-36.2) in the most recent period. In all periods the proportion of those stating the use of condoms for contraception who also reported its use at most recent coitus was high, ranging from 84 to 90%.

**Table 3:** Median percentages of using condoms at most recent coitus, by period and current contraceptive use status

Not using any method			Condoms		Non-barrier methods	
Period	Median	(95%, CI)	Median	(95%, CI)	Median	(95%, CI)
1991–1995	NE		NE		NE	
1996–2000	7.9	(4.8, 12.4)	84.4	(77.3, 91.8)	15.3	(11.3, 23.9)
2001–2005	16.1	(9.5, 24.2)	84.1	(78.2, 92.7)	21.1	(16.3, 28.8)
2006–2010	31.5	(13.7, 34.1)	88.9	(83.3, 91.0)	21.9	(10.3, 46.2)
2011–2015	29.0	(17.8, 37.1)	88.2	(84.5, 95.5)	30.8	(27.4, 36.2)

*NE* Not Estimated due to few number of surveys

[Denominator: single women aged 15-24 years, sexually active in the past 3 months].

We also examined the contraceptive status among women reporting condom use at most recent coitus (Additional file 8). The proportions using a non-barrier method of contraception were low in all periods but the proportions using no contraceptive method rose from 19.7% in 1996–2000 to 36.1% in 2011–15. The proportions reporting condom use for contraception fluctuated between 48 and 61%.

### Country ranking by behavioural risk

We defined behavioural risk as having sex in the past 3 months without protection from any contraceptive method nor use of a condom at last sex. The proportion among all single women aged 15-24 years defined thus as at risk are shown in the left-hand columns of Additional file 9 while the right-hand columns exclude virgins. When based on all women, which includes the protective contribution of virginity, the median percent at risk was 7.5%. It was below 2% in six countries and exceeded 20% in Liberia, Cote d’Ivoire and Sierra Leone. When based on sexually experienced women, the median rose to about 20%, with the highest values found in Chad, Liberia and Mali.

### Crude versus standardised results

Adjustment of results for age, education and residence makes surprisingly little difference (Table 1). With regards to abstinence, the two sets of estimates are closely similar, with the single exception of virginity in the most recent period, where the estimate of 58.4% (95%CI: 51. 7-68.6) was appreciable higher than the crude figure of 54.8% (95%CI: 49. 1-67.9), but did not attain statistical significance. For contraceptive use and condom use at most recent coitus, the sharpness of upward trends was only slightly attenuated.

## Discussion

The merit of this paper depends entirely on the credibility of reports by young African women on their behaviour. An extreme sceptic might claim that we have documented no more than the propensities of respondents to give socially desirable answers. We accept that the willingness of young single women to disclose sexual activity may well vary between countries. We checked the potential bias arising from this possibility by repeating the trend analysis for the sub-set of countries with 4 or more surveys but found very similar results to those based on all surveys. Reporting may also be affected by quality of interviewing and mode of data capture. We have minimized this type of uncertainty by relying on a single source, the DHS. The DHS is generally regarded as the gold standard for interview surveys in developing countries and the questions relevant to the topics of this paper are highly standardised across countries and over time.

Nevertheless, in the absence of any practical means of validating responses, interpretation of results needs to be cautious, particularly for reports of sexual behaviour; some country-specific trends are erratic and unconvincing. We are more confident about trends in use of contraceptives and condoms, which are more pronounced and regular than those for sexual abstinence.

We acknowledge a further limitation. The African countries included in this analysis are very diverse in terms of sexual norms, marriage customs, gender relations, disease environment and contraceptive practices. In presenting a regional overview, inevitably we could not do justice to this diversity. Country-specific results have been presented but it was beyond the scope of this paper to attempt any interpretation of varying trends.

Bearing in mind these caveats, what are the main findings and their implications? The 20 year time period covered in our analyses spans the rapid rise of HIV epidemics in many African countries, the peak incidence in the late 1990s and the subsequent fall in new infections. Despite the emphasis on abstinence of the US President’s Emergency Plan for AIDS Relief (PEPFAR) since the early years of this century, we see no evidence of radical changes in sexual behaviour, though it is entirely possible that numbers of sexual partners have fallen. The reported median level of primary abstinence, or virginity, which fluctuates between 55 and 62% is similar to levels observed in Latin America, USA, UK and Australia [15]. The high level of abstinence in the past 3 months among the sexually experienced, varying from 51 to 38% in different periods, is of particular interest because it implies low coital frequency, much lower than for cohabiting women and much lower than for single women in USA or UK [15]. Social disapproval of, or ambivalence about, sexual activity of single girls and young women in Africa is one factor constraining sexual encounters, which often need to be concealed, thus limiting opportunities for sexual encounters [16, 17]. Even after allowance for a degree of possible over reporting, primary and secondary abstinence remain the main way in which single African women protect themselves, as shown by the small proportions at risk in many countries when abstinence is taken into account. Infrequent sex also has implications for choice of contraceptive method, to be discussed below.

While exposure to intercourse has probably changed little over the past 20 years, reported current use of a modern contraceptive method and reported use of a condom at most recent coitus has doubled since the early 1990s among the sexually active. These trends do not reflect changes in the educational, residential and age composition of the study population. The key period of change for contraceptive use was the decade from the early 1990s to early 2000s, when a major and welcome shift from periodic abstinence to condoms occurred. It is of concern that further improvements in modern contraceptive protection since 2001–2005 have been minor. It is also surprising because of the increased commitment to international family planning, and the increased emphasis of the needs of adolescents, leading up to and following the 2012 London Summit on this subject.

Nevertheless, contrary to popular belief, modern method contraceptive use is considerably higher among sexually active single women than among married or cohabiting women, no doubt because of a higher level, and greater intensity, of desire to prevent pregnancy among the single than the married [18]. Method choice also differs radically. Among married African women, injectables are the dominant method, accounting for nearly half of all use, though implants are increasingly popular; condoms are rarely used within marriage except in Southern African countries with particularly severe HIV epidemics [19]. In contrast, the condom is the major method among young single women, though its contribution to overall protection has slipped from 61 to 51% in the past decade. It is encouraging that close to 90% of those reporting condom use as their contraceptive method also reported use of this method at last coitus as this indicator is highly correlated with consistent use [20, 21]. Over the 20 year period, the contribution of orals and injectables to protection has increased from 10 to 24% but use of IUDs and implants remains trivial.

Condom use at most recent coitus was ascertained later in the interview from questions on contraception and made no mention of motive for use. Among those who reported use at last coitus, about half had previously claimed current use of this method for pregnancy-prevention. While the relative importance of the two motives for condom use—prevention of pregnancy and disease—is very difficult to establish, our findings suggest that a contraceptive motive is implicated for about half of all condom users. Double-method protection, simultaneous use of non-barrier contraception and condoms, is commonly regarded as ideal but is a rare form of behaviour [22]. We found encouraging results in that regard: in the most recent period, 31% of users of a non-barrier method reported condom use at last coitus, a level of use similar to that among non-users of contraception.

These findings raise fundamental questions about optimal protective strategies for sexually active young single women in Africa, particularly in settings where risk of infection with HIV or classical sexually transmitted diseases is high. Should we welcome the recent observed shift away from condoms to oral and injectable contraceptives and the strategic emphasis, so far apparently unsuccessful, on encouraging the use of long-acting methods for adolescents [23]? The strongest justification for hormonal method use by single women is that their use does not depend on the cooperation of male partners. Particularly when the male partner is much older, a young woman’s urgency to enforce the use of a condom may be weak. It is also true that consistent condom use requires more foresight and discipline than non-coital methods and becomes more problematic as relationships develop and the frequency of sex increases [24]. Clearly, no single method can meet the diverse needs and circumstances of sexually active young women and, of course, ultimately their freedom to choose must be respected.

Another common reason for welcoming increased use of hormonal methods by single women, that they are more effective than condoms, is less well founded. The failure rates of condoms and oral contraceptives are very similar both in East and West Africa [25]. Condom failure rates also tend to be lower for single than married women, probably because of differences in coital frequency [25]. Injectable users have lower failure rates but discontinuation of this method because of side effects and health concerns is high in married women and may be even higher in single women [26]. Moreover, the recognition by the World Health Organization that injectable use may increase the risks of HIV acquisition, and the consequent change in the Medical Eligibility Criteria, could limit future acceptability [27]. Failure and discontinuation rates for highly effective long-acting methods such as implants are low and this method is suitable for single women in countries such as USA and UK because of the long gap between sexual initiation and cohabitation or marriage and the relatively low risk of sexually transmitted infections. In Africa, the need for long term contraceptive protection from implants (or intrauterine devices) is lower than in USA or UK because marriage or cohabitation, and the desire for a child, typically occurs at young ages. We acknowledge that implants are well suited to the needs of young African women in committed long term, albeit non-cohabiting, relationships. In such circumstances, double method protection (ie implant together with condom use) remains the ideal.

From a pregnancy-prevention perspective, however, the condom remains the most suitable option for the large number, probably a majority, of young women who are not in a committed long term relationship and for whom coitus is infrequent. For them, permanent protection from daily use of oral contraceptives, injectables or implants probably seems excessive. The advent of emergency contraception and the rapid proliferation of medical abortion provide a back-up for occasions when condoms, for whatever reason, are not used or break during use.

The recent neglect of condoms by both the family planning and HIV communities, in our judgment, is regrettable. From a disease-prevention perspective, frequent HIV testing and treatment, pre-exposure prophylaxis for very high risk individuals, and male circumcision can make valuable contributions. Yet, condoms remain the major, and most cost effective, component of prevention [28, 29].

In our view the ideal contraceptive package for many young women comprises condoms, emergency contraceptives, pregnancy-testing kits, and advice, where legally possible, on medical abortion. A large majority of young people who use condoms obtain supplies from commercial sources [30]. Thus we urge a re-vitalisation of condom social marketing. The evidence on the effectiveness of mass media promotion and condom social marketing is generally positive [31, 32]. We further suggest that social marketing may be more successful with a pregnancy-prevention emphasis than with an HIV prevention one, for two reasons. First, the association of condoms with promiscuity and disease limits its use [33]. Second, it is obviously easier for a young woman to negotiate use to prevent pregnancy than to prevent HIV.

## Conclusions

Our paper complements the extensive existing literature in several ways. We believe that this paper presents the clearest chronological record of changes in key protective behaviours in young single women, thus far published. Drawing on all 112 surveys in sub-Saharan Africa that used the same protocol we document trends at regional and country level over a 20 year period. While trends in contraceptive use and condom use are well known, joint analyses of both outcomes are rare and most omit consideration of abstinence. We pay equal attention to all three behaviours, including shifts in type of contraceptive method used. Questions on contraception and on condom use at most recent coitus are posed at different times in the interview and thus may be regarded as semi-independent items of information. This allows us to measure the extent of dual protection and to estimate the approximate contribution of a contraceptive motive to overall condom use. Lastly, we estimate the effect of structural shifts in the study population, namely age, education and rural-urban residence, on reported behavioural change.

The protective role of sexual abstinence has not changed but remains strong. The high level of abstinence in the past 3 months among sexually experienced women indicated that coitus is infrequent for many. Mixed messages can be drawn from the analysis of trends in current contraceptive use and condom use at most recent coitus. In the past 20 years levels of both indicators have doubled but the pace of change has slowed in the past decade. The central policy lesson concerns the continuing centrality of the condom, both for prevention of pregnancy and disease. Condoms remain the dominant contraceptive method for single women in Africa and pregnancy-prevention is at least part of the motive for half of those reporting its use at last coitus. In our view, the condom is the optimal choice for women who have infrequent sex and live in settings where HIV risk is high. The advent of emergency contraception and medical abortion as back-ups further justifies use of this method. Displacement of condoms by promotion of hormonal methods of contraception for this population is a danger. Rather we urge a re-invigoration of condom social marketing with a strong contraceptive emphasis. Together with a continued HIV testing and treatment and promotion of male circumcision, such re-invigoration should further accelerate the decline in HIV incidence in African youth.

## Additional files

Additional file 1: Analysis population (unweighted numbers), by survey, fieldwork date and period. The year of fieldwork, the analysis period, the number of all women (aged 15–49), number of young (aged 15–24), number of young and single, number of young, single and ever had sex, number of young, single and had sex in last 3 months, number of young, single and had sex in last 3 months, and currently using modern contraceptives, and number of young, single and used condom in last coitus. (XLS 37 kb)

Additional file 2: Comparisons of the 4 outcomes between all countries and countries with 4+ surveys. The results of the sensitivity analysis comparing the rates of Primary abstinence, Secondary abstinence, Current use of modern contraception, and Condom use last sex between all countries and countries with 4 and more surveys. (XLS 30 kb)

Additional file 3: Trends in Primary and Secondary Abstinence (crude and adjusted) within country trends in primary and secondary abstinence, and mean annual rate of change. (XLS 48 kb)

Additional file 4: Correlation between education level (secondary or higher) and current use of modern methods at national level, All single women aged 15–24 who had sex in the last 3 months. Pearson correlation coefficients of education and current use of modern methods with *p*-values, by analysis period. (XLS 25 kb)

Additional file 5: Trends in current use of modern contraception and condom use last coitus. Trends in crude and adjusted Current use of modern contraception and condom use last coitus, mean annual rate of change with (*p*-values), by country. (XLS 47 kb)

Additional file 6: Correlation between percent condom use last coitus and HIV prevalence among 15–24 years old, by sex and period. Pearson correlation coefficients of condom use last coitus and HIV prevalence with p-values, by analysis period. (XLS 26 kb)

Additional file 7: Percent reporting consistent condom use with last partner, among women who used a condom at most recent coitus. Lower quartile, median, upper quartile and IQR of consistent condom use with last sexual partner, among Single women aged 15-24 years, sexually active in the past 3 months and reported condom use last coitus. (XLS 25 kb)

Additional file 8: Median percentages of current contraceptive use among those who reported condoms use at most recent coitus, by period and method type. Median percentages of current contraceptive use among those who reported condoms use at most recent coitus, by period and method type, among Single women aged 15–24 years, sexually active in the past 3 months and reported condom use last coitus. (XLS 25 kb)

Additional file 9: Ranking of countries according to risk among women aged 15-24. Percentage at risk among all women and women who had sex aged 15-24. (XLS 30 kb)
